# Supplementary material for: Gut microbiome composition and metabolic activity in women with diverticulitis
Source: Nat Commun. 2024 Apr 29;15:3612. doi: 10.1038/s41467-024-47859-4 (PMC11059386; doi:10.1038/s41467-024-47859-4)
Supplement: Supplementary file 1 — Supplementary Information [file 41467_2024_47859_MOESM1_ESM.pdf]

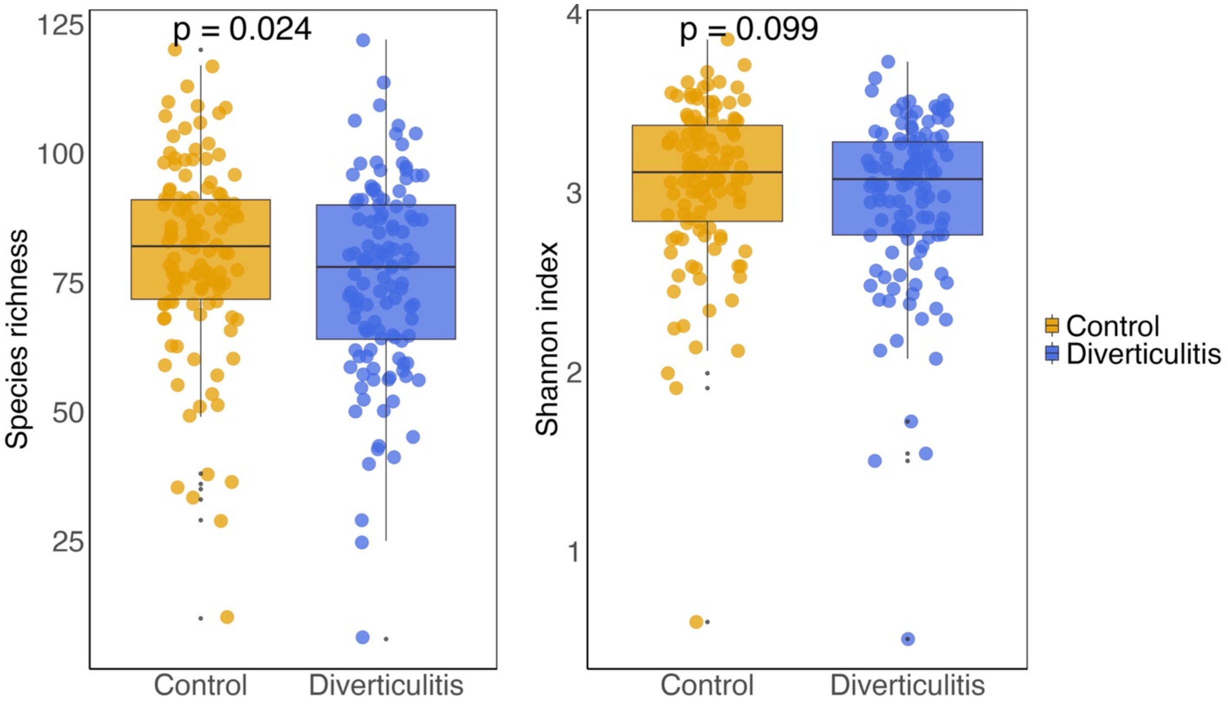

Supplementary Figure 1: Alpha-diversity indexes in diverticulitis and control. Patients with diverticulitis had a significant reduction in the species richness and a non-significant reduction in the Shannon index.

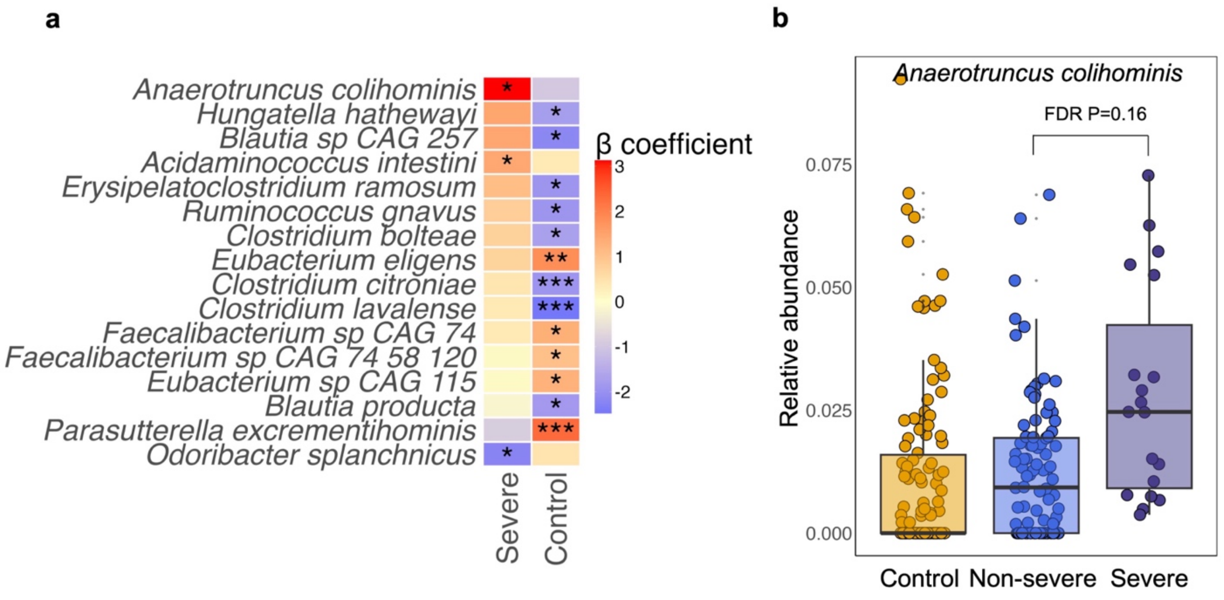

Supplementary Figure 2: Species abundances statistically significantly altered in severe diverticulitis that required surgery or had an abscess. a) β coefficients of the statistically significant associations of species with severe diverticulitis and control (non-severe diverticulitis was treated as the reference group). N=118, 98, 19, respectively for control, non-severe diverticulitis, and severe diverticulitis. We adjusted for age, race, Bristol stool scale, antibiotics use, fiber intake, alcohol consumption, body mass index, smoking, menopausal hormone use, physical activity, Alternate Healthy Eating Index, and calorie intake. P-values are two-sided, with multiple comparison corrected using Benjamini-Hochberg false discovery rate (FDR). **** $p<0.01$; *** $0.01<p<0.05$; ** $0.05<p<0.1$; * $0.1<p<0.25$. Complete results for associations between species and severity of diverticulitis from MaAsLin 2 models are provided in Supplementary Table 3. b) *Anaerotruncus colihominis* significantly increased in severe diverticulitis.

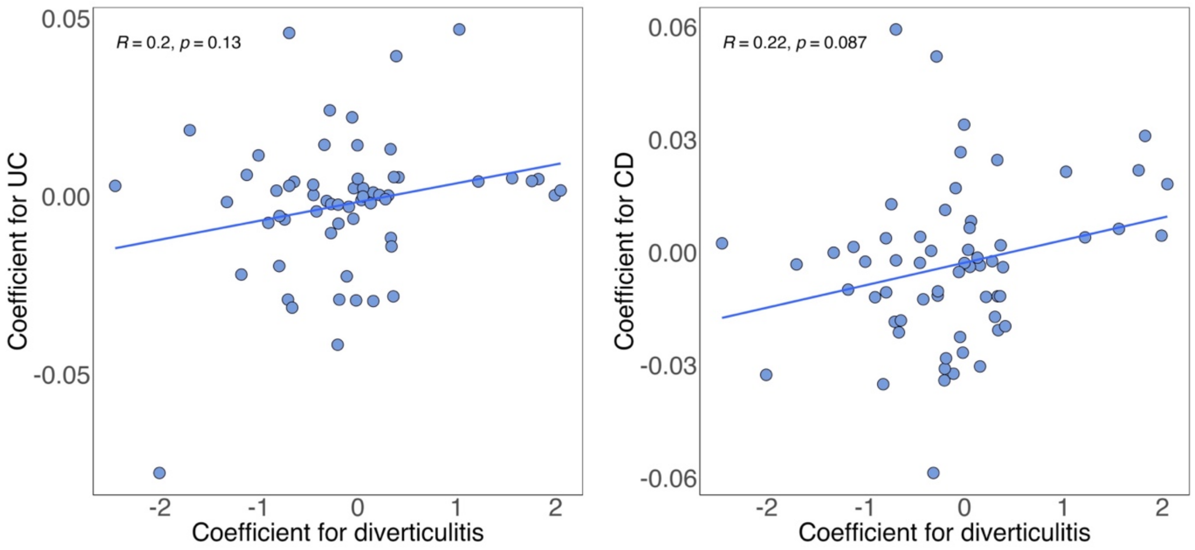

Supplementary Figure 3: Taxonomic changes in diverticulitis were similar to those observed in IBD. Coefficients for species and diverticulitis associations from multivariate testing were modestly correlated with those for ulcerative colitis and Crohn's disease reported in the Human Microbiome Project (HMP2). Data are provided in Supplementary Table 4.

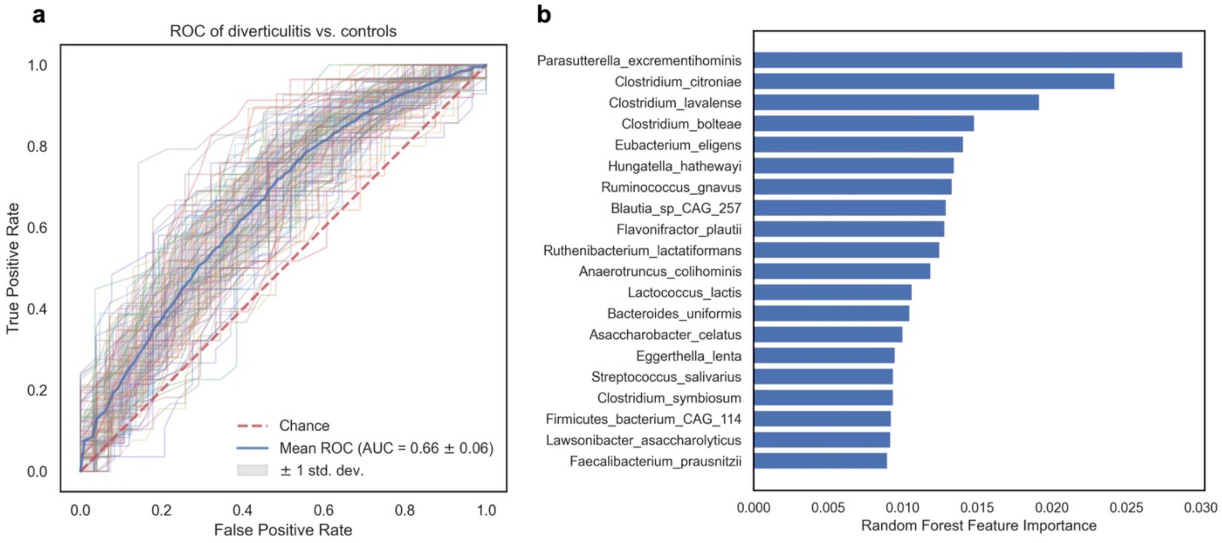

Supplementary Figure 4: Predictive value of the gut microbiome in the risk of diverticulitis. a) Discriminative classification of diverticulitis vs. controls using 189 microbial species in Random Forest, as implemented in the *scikit-learn* package in Python. The model was fit with cross-validation based on 100 random splits (*ShuffleSplit* in Python) and a 75/25 random split of training and testing folds. The performance of the random forest classifier was quantified by calculating the areas under the Receiver Operating Characteristics curve (AUC) over 100 iterations. b) We identified the top 20 microbial species contributing to the classification via the Gini importance (mean decrease impurity) from the random forest model.

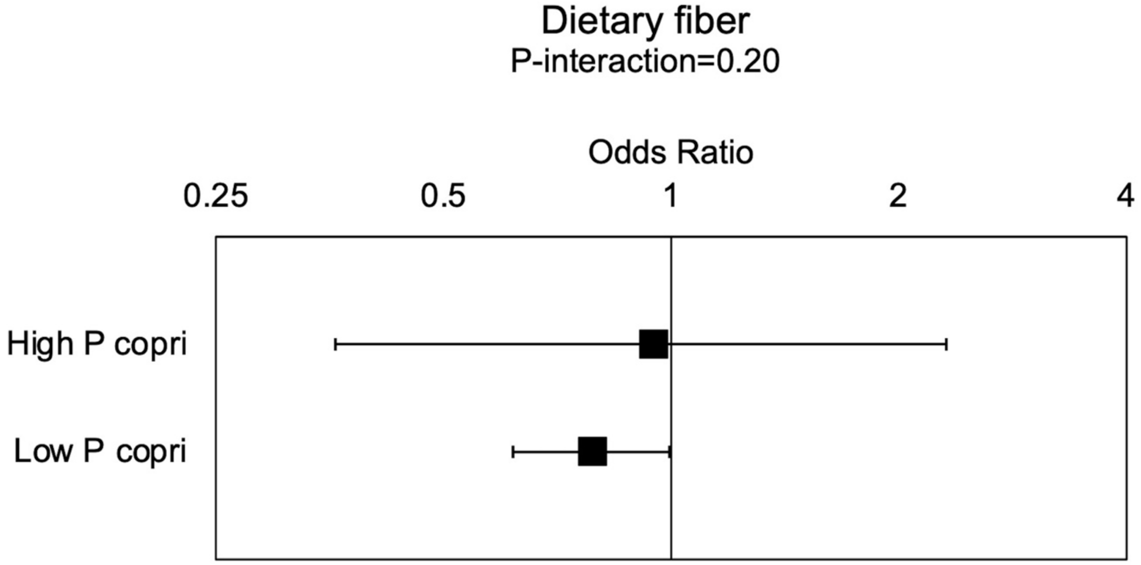

Supplementary Figure 5: P*revotella copri* modulates the association between dietary fiber intake and diverticulitis. We evaluated the associations between fiber intake and diverticulitis in subgroups according to carriage of *P. copri* (yes or no). We adjusted for age, race, Bristol stool scale, antibiotics use, alcohol consumption, body mass index, smoking, menopausal hormone use, physical activity, and calorie intake. Values represent odds ratios and 95% confidence intervals associated with each 5g increase in dietary fiber. P-interaction was assessed using a Wald test of the product term of fiber and abundance of *P. copri*. Higher fiber intake was associated with a greater reduction in the risk of diverticulitis among individuals who did not carry *P. copri*.

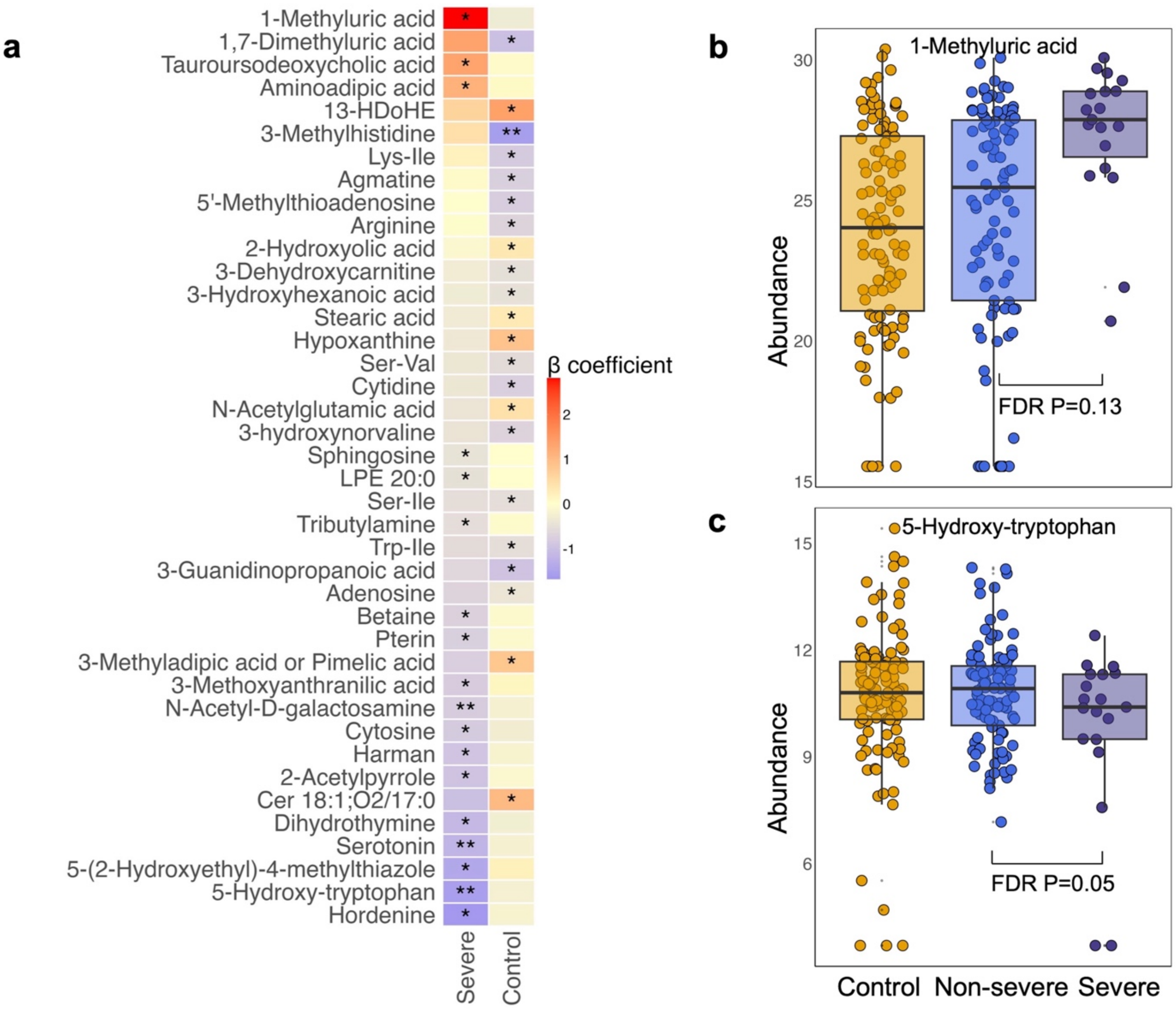

Supplementary Figure 6: Metabolites statistically significantly altered in severe diverticulitis that required surgery or had an abscess. a) β coefficients of the statistically significant associations of metabolites with severe diverticulitis and control (non-severe diverticulitis was treated as the reference group). We adjusted for age, race, Bristol stool scale, antibiotics use, fiber intake, alcohol consumption, body mass index, smoking, menopausal hormone use, physical activity, Alternate Healthy Eating Index, and calorie intake. P-values are two-sided, with multiple comparison corrected using Benjamini-Hochberg false discovery rate (FDR). **** $p<0.01$; *** $0.01<p<0.05$; ** $0.05<p<0.1$; * $0.1<p<0.25$. Complete results for associations between metabolites and severity of diverticulitis from MaAsLin 2 models are provided in Supplementary Table 11. b) 1-Methyluric acid increased in severe diverticulitis. c) 5-hydroxy-tryptophan reduced in severe diverticulitis.

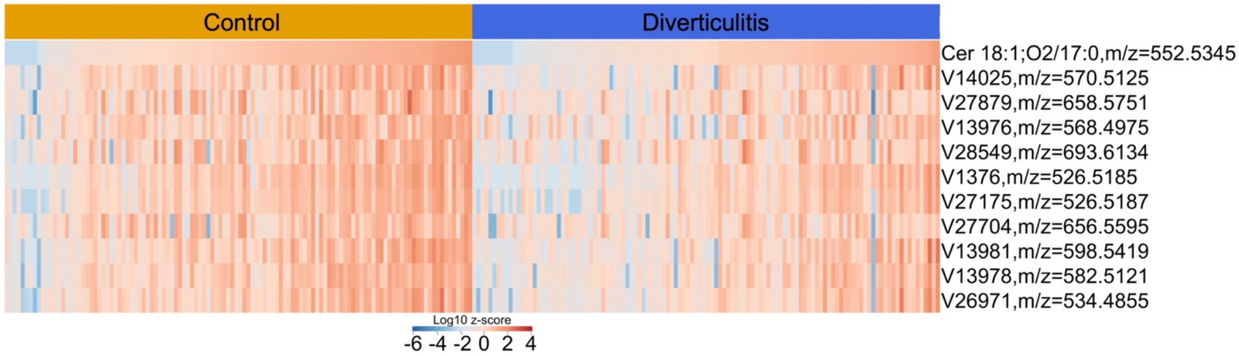

Supplementary Figure 7: A group of unannotated metabolites that covary with Cer(d18:1/17:0) were highly prioritized in diverticulitis. Abundances of the top prioritized metabolomic module associated with diverticulitis in MACARRoN, with adjustment for age, race, Bristol stool scale, antibiotics use, fiber intake, alcohol consumption, body mass index, smoking, menopausal hormone use, physical activity, Alternate Healthy Eating Index, and calorie intake. Values were log10 transformed and z-score normalized. Complete results for metabolomic features prioritized in diverticulitis from MACARRoN are provided in Supplementary Table 12.

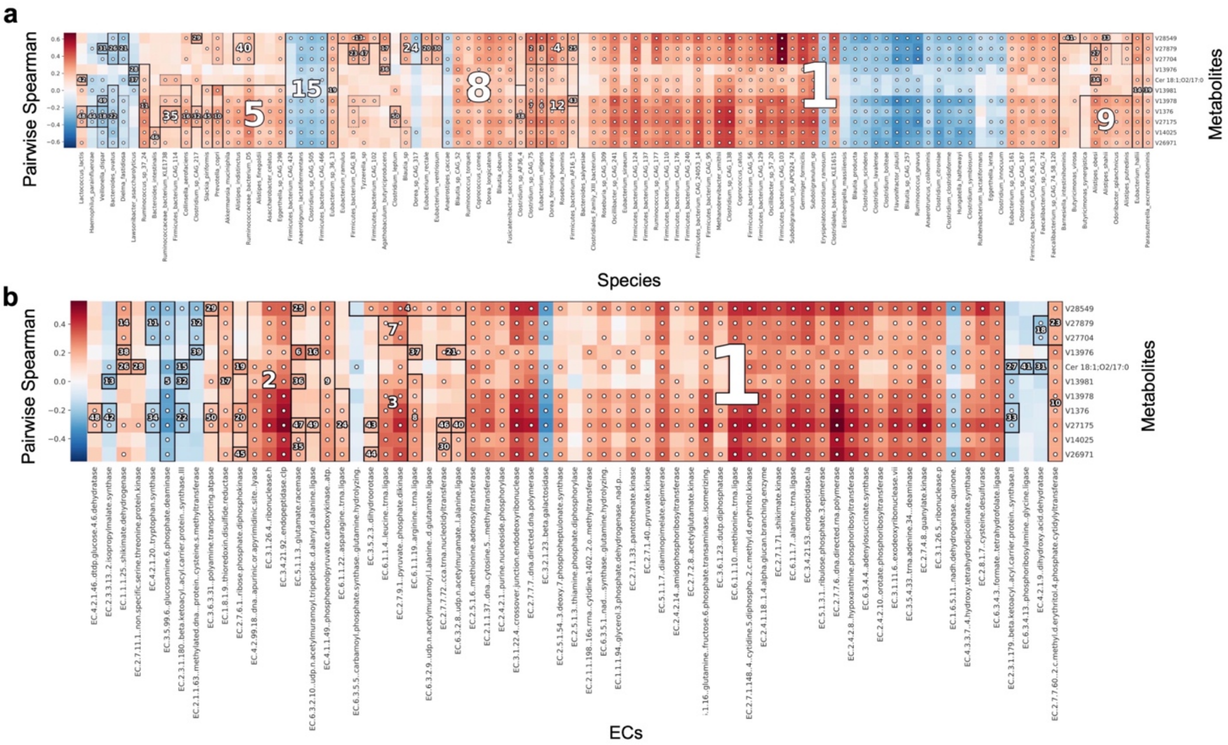

Supplementary Figure 8: Group of unannotated metabolites that covary with Cer(d18:1/17:0) and highly prioritized in diverticulitis corresponded to microbial composition and functional potentials. HAllA was used to discover many significant clusters of associations between this group of unknown metabolomic features and microbial species and functional potentials, including a) positive correlations with *Firmicutes bacterium* and *Subdoligranulum* but inverse correlations with *Ruminococcus gnavus* and *Clostridium* pathogens; and b) positive correlation with 1,4-alpha-glucan branching enzyme (EC 2.4.1.18) but inverse correlation with beta-galactosidase (EC 3.2.1.23). We restricted to the 100 most abundant and independent (spearman r<=0.7) ECs using orthogonal selection.

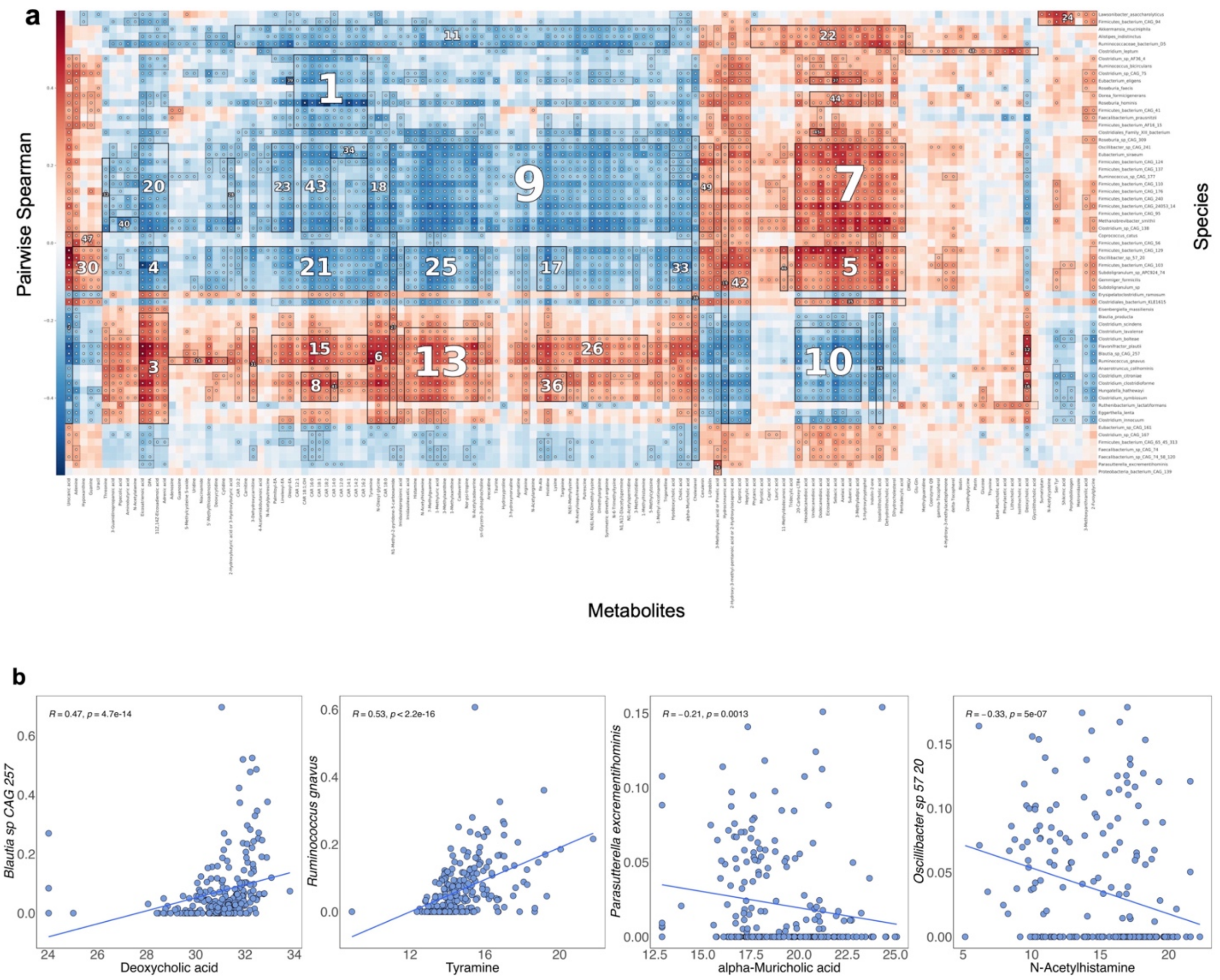

Supplementary Figure 9: Significant associations between microbial species and metabolites across the whole samples. a) HAllA plot showing the clusters of related associations (blocks) between species and metabolites. b) Examples of biologically plausible associations between species and metabolite pairs.
