# Supplementary material for: Gut microbiome composition and metabolic activity in women with diverticulitis
Source: Nat Commun. 2024 Apr 29;15:3612. doi: 10.1038/s41467-024-47859-4 (PMC11059386; doi:10.1038/s41467-024-47859-4)
Supplement: Supplementary file 3 — Description of Additional Supplemental Data [file 41467_2024_47859_MOESM3_ESM.docx]

Description of Additional Supplementary Files

File Name: Supplementary Data 1

Description: PERMANOVA of Bray-Curtis dissimilarities of metabolomics profile explained by the microbiome, diverticulitis, and other factors.

File Name: Supplementary Data 2

Description: Associations between species abundances and diverticulitis. Multivariate model in MaAsLin 2 was adjusted for age, race, Bristol stool scale, antibiotics use, fiber intake, alcohol consumption, body mass index, smoking, menopausal hormone use, physical activity, Alternate Healthy Eating Index, and calorie intake. P-values are two-sided, with multiple comparison corrected using Benjamini-Hochberg false discovery rate (FDR).

File Name: Supplementary Data 3

Description: Associations between species abundances and severity of diverticulitis. Multivariate model in MaAsLin 2 was adjusted for age, race, Bristol stool scale, antibiotics use, fiber intake, alcohol consumption, body mass index, smoking, menopausal hormone use, physical activity, Alternate Healthy Eating Index, and calorie intake. P-values are two-sided, with multiple comparison corrected using Benjamini-Hochberg false discovery rate (FDR). Non-severe diverticulitis was treated as the reference group.

File Name: Supplementary Data 4

Description: Coefficients for species in association with diverticulitis as compared to those for Crohn's disease and ulcerative colitis in HMP2.

File Name: Supplementary Data 5

Description: Associations between metagenomic pathways and diverticulitis. Multivariate model in MaAsLin 2 was adjusted for age, race, Bristol stool scale, antibiotics use, fiber intake, alcohol consumption, body mass index, smoking, menopausal hormone use, physical activity, Alternate Healthy Eating Index, and calorie intake. P-values are two-sided, with multiple comparison corrected using Benjamini-Hochberg false discovery rate (FDR).

File Name: Supplementary Data 6

Description: Associations between metagenomic functionals and diverticulitis. Multivariate model in MaAsLin 2 was adjusted for age, race, Bristol stool scale, antibiotics use, fiber intake, alcohol consumption, body mass index, smoking, menopausal hormone use, physical activity, Alternate Healthy Eating Index, and calorie intake. P-values are two-sided, with multiple comparison corrected using Benjamini-Hochberg false discovery rate (FDR).

File Name: Supplementary Data 7

Description: Associations of dietary fiber and major dietary patterns with risk of diverticulitis. Multivariate model was adjusted for age, race, body mass index, physical activity, calorie intake, menopausal hormone use, and alcohol consumption; Multivariate+diet model was additionally adjusted for dietary intake of fiber (except for models assessing fiber as an exposure) and red/processed meat. P-trend was assessed by including dietary fiber and patterns as continuous variables.

File Name: Supplementary Data 8

Description: Species loadings to the first two principal coordinates of Bray-Curtis dissimilarities.

File Name: Supplementary Data 9

Description: Metabolite subclasses that were significantly altered in diverticulitis.

File Name: Supplementary Data 10

Description: Associations between metabolites and diverticulitis. Multivariate model in MaAsLin 2 was adjusted for age, race, Bristol stool scale, antibiotics use, fiber intake, alcohol consumption, body mass index, smoking, menopausal hormone use, physical activity, Alternate Healthy Eating Index, and calorie intake. P-values are two-sided, with multiple comparison corrected using Benjamini-Hochberg false discovery rate (FDR).

File Name: Supplementary Data 11

Description: Associations between metabolites and severity of diverticulitis. Multivariate model in MaAsLin 2 was adjusted for age, race, Bristol stool scale, antibiotics use, fiber intake, alcohol consumption, body mass index, smoking, menopausal hormone use, physical activity, Alternate Healthy Eating Index, and calorie intake. P-values are two-sided, with multiple comparison corrected using Benjamini-Hochberg false discovery rate (FDR). Non-severe diverticulitis was treated as the reference group.

File Name: Supplementary Data 12

Description: Top-ranked prioritized metabolite module associated with diverticulitis in MACARRoN.

File Name: Supplementary Data 13

Description: Microbe-metabolite relationships in diverticulitis and controls.
