# Supplementary material for: Gut microbiome composition and metabolic activity in women with diverticulitis
Source: Nat Commun. 2024 Apr 29;15:3612. doi: 10.1038/s41467-024-47859-4 (PMC11059386; doi:10.1038/s41467-024-47859-4)
Supplement: Supplementary file 5 — Reporting Summary [file 41467_2024_47859_MOESM5_ESM.pdf]

nature portfolio

Corresponding author(s): Andrew T. Chan

Last updated by author(s): Apr 1, 2024

Reporting Summary

Nature Portfolio wishes to improve the reproducibility of the work that we publish. This form provides structure for consistency and transparency in reporting. For further information on Nature Portfolio policies, see our Editorial Policies and the Editorial Policy Checklist.

Statistics

For all statistical analyses, confirm that the following items are present in the figure legend, table legend, main text, or Methods section.

n/a	Confirmed	
☐	☒	The exact sample size (*n*) for each experimental group/condition, given as a discrete number and unit of measurement
☐	☒	A statement on whether measurements were taken from distinct samples or whether the same sample was measured repeatedly
☐	☒	The statistical test(s) used AND whether they are one- or two-sided<br>*Only common tests should be described solely by name; describe more complex techniques in the Methods section.*
☐	☒	A description of all covariates tested
☐	☒	A description of any assumptions or corrections, such as tests of normality and adjustment for multiple comparisons
☐	☒	A full description of the statistical parameters including central tendency (e.g. means) or other basic estimates (e.g. regression coefficient) AND variation (e.g. standard deviation) or associated estimates of uncertainty (e.g. confidence intervals)
☐	☒	For null hypothesis testing, the test statistic (e.g. *F*, *t*, *r*) with confidence intervals, effect sizes, degrees of freedom and *P* value noted<br>*Give P values as exact values whenever suitable.*
☒	☐	For Bayesian analysis, information on the choice of priors and Markov chain Monte Carlo settings
☒	☐	For hierarchical and complex designs, identification of the appropriate level for tests and full reporting of outcomes
☐	☒	Estimates of effect sizes (e.g. Cohen's *d*, Pearson's *r*), indicating how they were calculated

Our web collection on statistics for biologists contains articles on many of the points above.

Software and code

Policy information about availability of computer code

Data collection	Metagenomic and metabolomic profiling conducted at the Broad Institute<br>Metagenomic profiles generated using bioBakery3 meta'omics workflow (Preprocessing: KneadData 0.12.0; Taxonomic profiles: MetaPhlAn3 3.1.0; Functional profiles: HUMAnN 3.5)<br>Metabolomic data generated using a combination of four LC–MS methods (HILIC-pos, HILIC-neg, C18-neg, and C8-pos)
Data analysis	R 4.2.1 (main packages: vegan 2.6-4, MaAsLin2 1.10.0, MACARRoN, glm)<br>HAllA 0.8.20<br>Python (scikit-learn 1.0.2)<br>All codes have been deposited in GitHub repository (https://github.com/biobakery/MicroN-diverticulitis)

For manuscripts utilizing custom algorithms or software that are central to the research but not yet described in published literature, software must be made available to editors and reviewers. We strongly encourage code deposition in a community repository (e.g. GitHub). See the Nature Portfolio guidelines for submitting code & software for further information.

Data

Policy information about availability of data

All manuscripts must include a data availability statement. This statement should provide the following information, where applicable:

- Accession codes, unique identifiers, or web links for publicly available datasets
- A description of any restrictions on data availability
- For clinical datasets or third party data, please ensure that the statement adheres to our policy

Metagenomic sequencing data and raw metabolomics data have been deposited to dbGaP (study accession: phs003531.v1.p1). Access to individual-level data is granted upon approval of a data access request to dbGaP: https://view.ncbi.nlm.nih.gov/dbgap-controlled.

Research involving human participants, their data, or biological material

Policy information about studies with human participants or human data. See also policy information about sex, gender (identity/presentation), and sexual orientation and race, ethnicity and racism.

Reporting on sex and gender	Our study was conducted within a cohort that only enrolled female registered nurses, thus we are able to report findings only in one sex. We have explicitly described this in abstract and the manuscript.
Reporting on race, ethnicity, or other socially relevant groupings	We have collected information on race/ethnicity. We matched diverticulitis cases and controls based on race and controlled for it in the analyses.
Population characteristics	Participants included 121 women who reported a diagnosis of diverticulitis requiring antibiotics or hospitalizations from the 2019 biennial cohort questionnaire with each matched to a diverticulitis-free controls according to age, race, and month of stool collection. The mean age of participants was 65 years old. Characteristics of the study population were described in detail in the manuscript.
Recruitment	We conducted a nested case-control study within the Nurses' Health Study (NHS) II, which is an ongoing prospective cohort of 116,429 female registered nurses residing across the U.S. aged 25-42 years at enrollment in 1989. Participants have been followed biennially by querying lifestyle, medical or other health-related information. Beginning in February 2019, a large-scale prospective collection of fecal microbiome samples was launched for NHS II participants, known as the Microbiome among Nurses (Micro-N) project. As of July 2021, a total of 14,992 women have returned a collection kit. Among these participants, we ascertained 121 incident diverticulitis requiring antibiotics or hospitalization based on information reported in the 2019 biennial questionnaire and matched each of the case to a diverticulitis-free control.
Ethics oversight	The study was approved by Institutional Review Boards of Brigham and Women's Hospital and Harvard T.H. Chan School of Public Health.

Note that full information on the approval of the study protocol must also be provided in the manuscript.

Field-specific reporting

Please select the one below that is the best fit for your research. If you are not sure, read the appropriate sections before making your selection.

☒ Life sciences ☐ Behavioural & social sciences ☐ Ecological, evolutionary & environmental sciences

For a reference copy of the document with all sections, see nature.com/documents/nr-reporting-summary-flat.pdf

Life sciences study design

All studies must disclose on these points even when the disclosure is negative.

Sample size	Among 14,992 women who have returned a stool sample as of July 2021, we identified 121 women who reported a diagnosis of diverticulitis requiring antibiotics or hospitalization on the 2019 cohort questionnaire and matched each of the case to a diverticulitis-free control (total N=242).
Data exclusions	One sample with no reads detected was excluded from further analysis.
Replication	The study was a large-scale population-based cohort study and we did not attempt to replicate all aspects of sample collection and data generation. However, data and source code used are available to the public and therefore all of our analyses can be reproduced using our methods or re-analyzed using other methods. When possible, we refer to existing literature that supports our findings.
Randomization	This study was a case-control study nested within a prospective cohort. There was no intervention allocated so randomization was not relevant. However, where able, we did match diverticulitis cases and controls on key factors (age, race, and month of stool collection) and adjusted for a list of covariates to minimize the potential for confounding.
Blinding	Blinding was not relevant to this study, given there was no intervention.

Reporting for specific materials, systems and methods

We require information from authors about some types of materials, experimental systems and methods used in many studies. Here, indicate whether each material, system or method listed is relevant to your study. If you are not sure if a list item applies to your research, read the appropriate section before selecting a response.

Materials & experimental systems

n/a	Involved in the study
☒	☐ Antibodies
☒	☐ Eukaryotic cell lines
☒	☐ Palaeontology and archaeology
☒	☐ Animals and other organisms
☒	☐ Clinical data
☒	☐ Dual use research of concern
☒	☐ Plants

Methods

n/a	Involved in the study
☒	☐ ChIP-seq
☒	☐ Flow cytometry
☒	☐ MRI-based neuroimaging

Plants

Seed stocks

Report on the source of all seed stocks or other plant material used. If applicable, state the seed stock centre and catalogue number. If plant specimens were collected from the field, describe the collection location, date and sampling procedures.

Novel plant genotypes

Describe the methods by which all novel plant genotypes were produced. This includes those generated by transgenic approaches, gene editing, chemical/radiation-based mutagenesis and hybridization. For transgenic lines, describe the transformation method, the number of independent lines analyzed and the generation upon which experiments were performed. For gene-edited lines, describe the editor used, the endogenous sequence targeted for editing, the targeting guide RNA sequence (if applicable) and how the editor was applied.

Authentication

Describe any authentication procedures for each seed stock used or novel genotype generated. Describe any experiments used to assess the effect of a mutation and, where applicable, how potential secondary effects (e.g. second site T-DNA insertions, mosiacism, off-target gene editing) were examined.
